# Efficacy and safety of intrathecal dexamethasone combined with isoniazid in the treatment of tuberculous meningitis: a meta-analysis

**DOI:** 10.1186/s12883-024-03701-4

**Published:** 2024-06-10

**Authors:** Yan Gao, Jinwen Su, Yuxiang Ma, Yunliang Sun, Jiyong Cui, Xianhe Jin, Yuxi Li, Zhi Chen

**Affiliations:** 1 Experimental Research Center, China Academy of Chinese Medical Sciences, Beijing, 100700 China; 2 ICU, Tuberculosis Department, 8th Medical Center of Chinese PLA General Hospital Tuberculosis Research Institute, Beijing, 100091 China

**Keywords:** Tuberculous meningitis, Intrathecal injection, Isoniazid, Dexamethasone, Meta-analysis

## Abstract

**Background:**

The treatment regimen for tuberculous meningitis (TBM) remains unclear and requires optimization. There are some reports on successful adjunct intrathecal dexamethasone and isoniazid (IDI) treatment strategies for TBM, however, there is equivocal evidence on their efficacy and safety.

**Methods:**

A comprehensive search of English and Chinese databases was conducted from inception to February 2024. A meta-analysis was performed on randomized controlled trials (RCTs) estimating the effects of adjunct IDI on conventional anti-TB (C anti-TB) treatments or C anti-TB alone. Efficacy, adverse reaction rate, cerebrospinal fluid (CSF) leukocytes, and CSF protein were used as primary outcome indicators. CSF glucose, CSF chlorides, CSF pressure, recovery time for laboratory indicators and recovery time for clinical symptoms were used as secondary outcome indicators.

**Results:**

A total of 17 studies involving 1360 (IDI group vs. C anti-TB group: 392 vs. 372; higher-dose IDI group vs. lower-dose IDI group: 319 vs. 277) patients were included in our analysis. Efficacy was significantly higher (RR 1.3, 95% CI 1.2-1.4, *P* < 0.001) and adverse reaction rate was significantly lower in the IDI groups (RR 0.59, 95% CI 0.37-0.92, *P* = 0.021). Furthermore, CSF leukocytes (WMD - 29.33, 95% CI [- 40.64 to-18.02], *P* < 0.001) and CSF protein (WMD - 0.79, 95%CI [-0.96 to-0.61], *P* < 0.001) were significantly lower in the IDI groups. Recovery time indicators were all shorter in the IDI groups, fever (SMD - 2.45, 95% CI [-3.55 to-1.35], *P* < 0.001), coma (SMD-3.75, 95% CI [-4.33 to-3.17], *P* < 0.001), and headache (SMD  - 3.06, 95% CI [- 4.05 to-2.07], *P* < 0.001), respectively. Higher-dose IDI was more effective than lower-dose IDI (RR 1.23, 95% CI 1.14-1.33, *P* < 0.001), with no significant difference in adverse reaction rate between the two (RR 0.82, 95%CI 0.43–1.56, *P* = 0.544).

**Conclusion:**

Adjunct IDI with C anti-TB can enhance therapeutic outcomes and reduce adverse reaction rate in adult TBM patients, with higher-dose IDI showing superior efficacy. These findings highlight the potential of IDI as an adjunctive therapy in TBM management. However, more high-quality RCTs from more regions should be conducted to support our results.

**Trial registration:**

Retrospectively registered in PROSPERO https://www.crd.york.ac.uk/prospero/display_record.php?ID=CRD42023388860.

**Supplementary Information:**

The online version contains supplementary material available at 10.1186/s12883-024-03701-4.

## Introduction

Tuberculous meningitis (TBM) accounts for 4.55% of all tuberculosis (TB) cases and is a rare complication of extra-pulmonary TB with high mortality rates [1]. The clinical presentation of TBM varies and initial symptoms, such as fever, headache, vomiting, and meningeal irritation signs, are similar to those of other types of meningitis, particularly cerebral toxoplasmosis, aspergillosis, neurosyphilis, and cryptococcal meningitis [2, 3], complicating early diagnosis. Current treatment strategies for TBM are unsatisfactory: there is no ‘gold standard’ rapid diagnostic test in current use and diagnosis is made using standard laboratory tests, e.g., microbiological, immunological, and biochemical tests, and nucleic acid amplification tests [4, 5]. Furthermore, drug-resistant TBM is widely reported, and current anti-TB drugs (e.g., isoniazid, rifampicin) have limited efficacy against drug-resistant TBM [6]. In addition, inadequate central nervous system (CNS) drug penetration of oral or intravenous anti-TB treatments (e.g., rifampicin, ethambutol) contributes to its high mortality rates [7]. For these reasons, TBM treatment outcomes remain poor [5, 8].

Currently, TBM is treated primarily using oral or intravenous anti-pulmonary TB regimens, the drug regimen and dosing duration for each stage of TBM are not as clearly defined compared with pulmonary TB [9], leading to urgent calls for action for new and better defined treatment strategies [4]. It has been suggested that intrathecal anti-TB medication has become a promising approach for the treatment of TBM [10], where drugs used for intrathecal injections include streptomycin and hydrocortisone [11], isoniazid, dexamethasone, and chymotrypsin [12], and isoniazid and prednisone [13]. It is also suggested that intrathecal antibiotics should be considered, especially for multi-drug-resistant bacteria, if no response to intravenous antibiotics or CSF drug concentrations is achieved [14], and there is increasing evidence of their efficacy [12, 13, 15].

Isoniazid and dexamethasone are commonly used intrathecal agents in the clinical treatment of TBM, isoniazid is a critical first-line agent with high bactericidal activity and high blood–brain barrier penetration [16, 17]. Corticosteroids, including dexamethasone and prednisolone, which have been shown to reduce inflammation, can improve clinical outcomes and survival rates for TBM [18–20]. The combination of intrathecal isoniazid with dexamethasone may offer synergistic effects that enhance the overall therapeutic efficacy.

Therefore, this meta-analysis aims to evaluate the current evidence for the efficacy and safety of adjunct IDI in adult TBM patients. This study will contribute to the optimization of TBM management protocols and potentially improve patient outcomes in this challenging condition.

## Methods

### Literature search

We conducted a comprehensive literature search of English and Chinese literature databases: PubMed, Web of Science, Embase, Cochrane, Chinese National Knowledge Infrastructure (CNKI), Wan Fang Database, Chinese Biomedical Literature Database (CBM), and the Chinese Scientific Journal Database (VIP) from inception to February 2024. Articles were retrieved using precise search terms and Boolean operators, details of which are provided in Additional file 1. The Preferred Reporting Items for Systematic Reviews and Meta-analysis (PRISMA) guidelines were followed [21].

### Inclusion and exclusion criteria

Randomized controlled trials (RCTs) on adult patients aged 18 and older with suspected or diagnosed TBM including the following two interventions:

1. Intrathecal dexamethasone and isoniazid + regular oral or intravenous anti-TB vs. regular oral or intravenous anti-TB.
2. Intrathecal higher concentration dexamethasone and isoniazid + regular oral or intravenous anti-TB vs. intrathecal lower concentration dexamethasone and isoniazid + regular oral or intravenous anti-TB.

All eligible studies must include at least one of the following primary outcome measures: treatment efficacy, adverse reaction rate, CSF leukocytes, and CSF protein concentration.

Exclusion criteria included the following:

1. Duplicate studies.
2. Conference proceedings or case reports.
3. Patients aged under 18.
4. Studies judged to be irrelevant during screening (i.e., by reading the title and abstract).
5. Non-RCTs (e.g., observational studies).
6. Studies reporting on patients with co-infections (e.g., HIV, fungal infections).
7. Studies where full datasets were unavailable in the publication or not provided precise data.

### Study selection

Study screening and selection were performed by two researchers (Y M & Y S), and when a decision could not be reached, a third researcher (J S) made the final decision.

### Data extraction and quality assessment

Data extraction was conducted independently by two researchers (J S & Y M). Data were tabulated in Excel (Microsoft Corp., Armonk, NY, USA) as follows:

1. Baseline details (authors, country, study period, publication year, total number of patients, study type, age, interventions (including drugs and doses), treatment time, and the frequency of Intrathecal injection).
2. Primary outcome measures
  - Treatment efficacy
  - Adverse reaction rate
  - CSF leukocytes
  - CSF protein concentrations
3. Secondary outcome measures
  - Secondary CSF findings (CSF glucose, chloride, and pressure)
  - Normalization of CSF findings and clinical signs and symptoms (CSF leukocytes, CSF protein, CSF pressure, fever, coma, and headache recovery times)

All data were cross-checked by (Y S & X J) and discrepancies were cross-checked by a third researcher (Z C).

The quality of the included studies was evaluated [22] and the following criteria were used: random sequence generation(i.e., whether subjects were randomly assigned to intervention and control groups); allocation concealment (i.e., whether investigators and participants were aware of the allocation scheme); blinding (i.e., whether participants, personnel administering interventions, and outcome assessors were aware of group assignments); incomplete outcome data and selective reporting (i.e., whether there was evidence of selective outcome reporting, particularly focusing on primary outcome measures). Full details on the quality evaluation are provided in Additional file 2.

### Statistical analysis

Dichotomous data were assessed using the pooled risk ratio (RR) with 95% confidence intervals (CIs), and continuous data were evaluated using the pooled weight mean difference (WMD) or standardized mean difference (SMD) with 95% CIs. Single-arm meta-analysis was performed for the adjunct IDI group, C anti-TB group, adjunct higher-dose IDI group, and adjunct lower-dose IDI group, respectively. Heterogeneity among studies was tested using the Cochran chi-squared test and I^2^ statistic, and when I^2^ > 50% this suggested significant heterogeneity and, in this case, a random-effects model was selected to gather the results, while a fixed-effects model was chosen when I^2^ < 50%. Publication bias was tested using the funnel plot test [23], Harbord test [24], Peters test [25], and Egger test [26]. For binary categorical variables, the Harbord and Peters tests were recommended, while the Egger test was recommended for numerical data. *P* < 0.5 was considered to indicate statistical significance (two-sided). We performed subgroup analyses based on the dose administered for the adjunct IDI groups. All statistical analyses were performed using STATA 12.0 (Stata Corp. LLC, College Station, TX, USA).

## Results

### Study selection and quality assessment

**Fig. 1:**
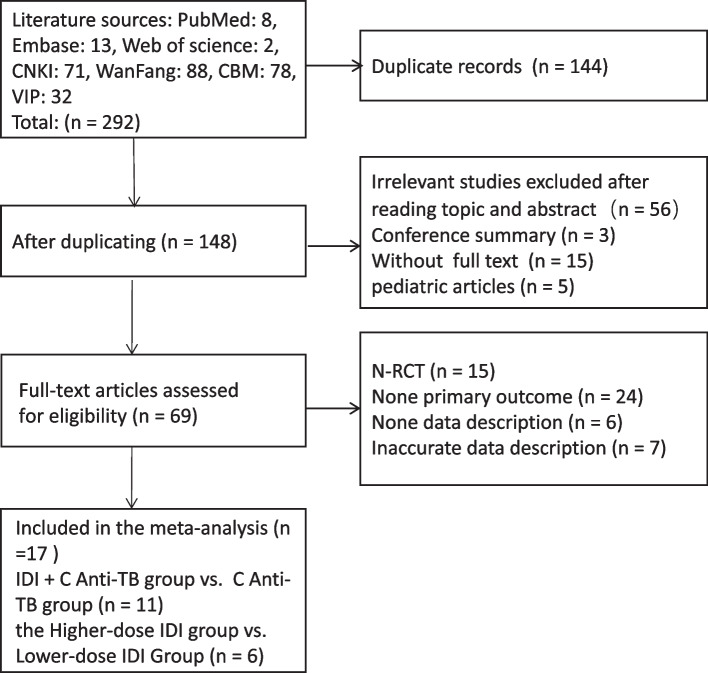
A flow diagram of the inclusion criteria of studies eligible for meta-analysis

According to the search strategy retrieved, 292 studies were obtained from the online literature databases from inception to February 2024. Following the removal of studies according to our inclusion and exclusion criteria. A total of 148 records were reserved after removing duplicates, and then 56 records were excluded after viewing the title and abstract, 3 conference papers, 5 pediatric articles, and 15 articles that were not available in the full text were also excluded. Here, we obtained 69 records that needed to be read carefully, and then 52 records were excluded for non-RCTs, with no primary outcomes, no data description, or inaccurate data description. Finally, 17 full-text primary studies were eligible included in this meta-analysis (Fig. 1). Of these, 11 were described intrathecal injection + conventional anti-TB in the test group, and 6 were described both intrathecal injection in the two groups, with a higher intrathecal injection dose in the test group than in the control group.

### Characteristics of the included studies

The characteristics of the included primary studies are provided in Tables 1 and 2.

**Table 1:** Characteristics of the included studies

**Study**	**Year**	**Country**	**IDI**	**C anti-TB**	**Study type**
Li et al. [27]	2017	China	Ii: INH100mg + DXM5mg	HRZS	RCT, non-blind
Shang et al. [28]	2017	China	Ii: INH50mg + DXM2mg	HRZE + DXM10mg/d	RCT, non-blind
Lu et al. [29]	2014	China	Ii: INH100mg + DXM3mg	HRZE(S) + DXM10 ~ 20 mg/d	RCT, non-blind
Chen et al. [30]	2010	China	Ii: INH100mg + DXM5mg	HRES	RCT, non-blind
Li et al. [31]	2012	China	Ii: INH50mg + DXM5mg	HRZE	RCT, non-blind
Bai et al. [32]	2020	China	Ii: INH100mg + DXM5mg	HRZE	RCT, non-blind
Yan et al. [33]	2015	China	Ii: INH50mg + DXM5mg	2HRZE/4HR + DXM10mg/d	RCT, non-blind
Wei et al. [34]	2010	China	Ii: INH50mg + DXM5mg	HRZE(S)	RCT, non-blind
He et al. [35]	2013	China	Ii: INH50mg + DXM3mg	3HRZE + 9HR	RCT, non-blind
Fan et al. [36]	2019	China	Ii: INH50mg + DXM3-5 mg	HRE	RCT, non-blind
Li et al. [37]	2021	China	Ii: INH100mg + DXM5mg	HRZE	RCT, non-blind
**Study**	**Year**	**Country**	**Higher-dose IDI**	**Lower-dose IDI**	**Study type**
Dilaremu et al. [38]	2020	China	Ii: INH100mg + DXM5mg	Ii: INH100mg + DXM2.5 mg	RCT, non-blind
Meng et al. [39]	2017	China	Ii: INH100mg + DXM5mg	Ii: INH50mg + DXM2.5 mg	RCT, non-blind
Jin et al. [40]	2019	China	Ii: INH100mg + DXM5mg	Ii: INH50mg + DXM2.5 mg	RCT, non-blind
Du et al. [41]	2016	China	Ii: INH200mg + DXM10mg	Ii: INH100mg + DXM5mg	RCT, non-blind
Zhang et al. [42]	2019	China	Ii: INH100mg + DXM5mg	Ii: DXM5mg	RCT, non-blind
Hu et al. [43]	2022	China	Ii: INH20mg + DXM5mg	Ii: INH20mg	RCT, non-blind

*INH* isoniazid, *DXM* dexamethasone, *Ii* intrathecal injection, *HRZE(S)* isoniazid (INH) + rifampicin (R) + pyrazinamide (PZA) + ethambutol (EMB) + streptomycin (Sm), *IDI* intrathecal dexamethasone, and isoniazid, *C anti-TB* conventional anti-tuberculosis, including oral or intravenous

The IDI group was conducted based on conventional anti-TB

**Table 2:** Characteristics of the included studies

**Study**	**Number (IDI /C anti-TB)**	**Total cases**	**Mean age (IDI / C anti-TB)**	**Treatment time (weeks)**	**The frequency of intrathecal injection (times/week)**
Li et al. [27]	47/46	93	33.51/34.21	12	2-3
Shang et al. [28]	20/20	40	39^a^	8	Once every 2 days
Lu et al. [29]	32/30	62	27.8/26.3	8	2-3
Chen et al. [30]	26/11	37	42^a^	8	Once every 2-4 days
Li et al. [31]	50/50	100	39.2/39.6	8	2-3
Bai et al. [32]	39/39	78	39.53/39.08	8	Once every 2 days
Yan et al. [33]	41/41	82	32.2/32.4	8	2-3
Wei et al. [34]	23/21	44	22^a^	8	2-3
He et al. [35]	34/34	68	32.7/31	8	#
Fan et al. [36]	46/46	92	39.46/39.57	8	2-3
Li et al. [37]	34/34	68	40.75/40.12	4	3
**Study**	**Number (higher-dose IDI / lower-dose IDI)**	**Total cases**	**Mean age (higher-dose IDI / lower-dose IDI)**	**Treatment time (weeks)**	The frequency of intrathecal injection (times/week)
Dilaremu et al. [38]	36/36	72	51.5/52.1	24	Not mentioned
Meng et al. [39]	70/40	110	45/45	24	Once every 2 days
Jin et al. [40]	60/60	120	67.3/66.3	12	1
Du et al. [41]	31/31	62	34.16/32.25	24	Once every (4 ± 1) days
Zhang et al. [42]	62/58	120	45.21/46.48	12	1
Hu et al. [43]	60/52	112	40.15/39.4	24	2-3

^a^Mean age of total participants in both the test and control groups

^#^Gradually reduce intrathecal injection frequency according to treatment outcome, usually stopping within 2 months

We performed meta-analyses of 11 (i.e., intrathecal injection + C anti-TB vs. C anti-TB) and 6 (i.e., a higher intrathecal injection dose vs. a lower intrathecal injection dose) studies, respectively.

### Results of IDI vs. C anti-TB therapy

#### Primary outcome indicators: efficacy, safety, and CSF findings

##### Treatment efficacy

**Fig. 2:**
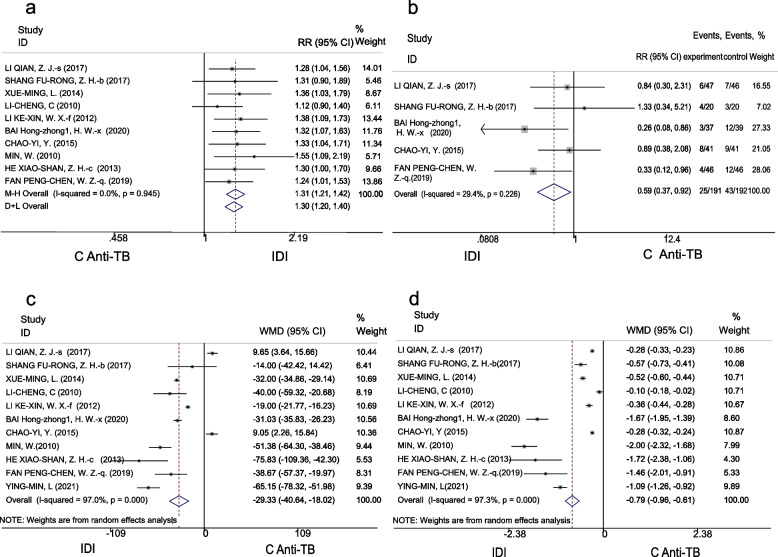
Forest plot of primary outcomes in the IDI and C anti-TB groups, (**a**) RR of the effective rate. **b** RR of the adverse reaction rate. **c** WMD of CSF leukocytes. **d** WMD of CSF protein concentration

The efficacy rates indicated 91% (95% CI 88% – 94%) and 70% (95% CI 65% – 75%) in the IDI and C anti-TB groups, respectively. Meta-analysis showed that treatment efficacy for patients receiving adjunct intrathecal injections was significantly higher than those receiving C anti-TB therapy alone (RR 1.3, 95% CI 1.2-1.4, *P* < 0.001). No heterogeneity was detected in the results (I^2^ = 0, *P* = 0.945) (Fig. 2a).

##### Adverse reaction rate

The adverse reaction rates in the IDI and C anti-TB groups were 12% (95% CI 7%-16%) and 21% (95% CI 15%-27%), respectively. Meta-analysis indicates that the adverse reaction rate of IDI was significantly lower than that among patients who received C anti-TB alone (RR 0.59, 95% CI 0.37-0.92, *P* = 0.021). There was no heterogeneity detected in the results (I^2^ = 29.4, *P* = 0.226) (Fig. 2b).

##### CSF leukocytes 

A random effects model was used to analyze CSF leukocytes due to high heterogeneity. The overall CSF leukocytes reported were 110.05 × 10^6^/L (95% CI 65.55–154.56) and 141.79 × 10^6^/L (95% CI 94.31–189.27) in the IDI and C anti-TB groups, respectively. The meta-analysis showed that CSF leukocytes were significantly lower in the IDI group than the C anti-TB group (WMD − 29.33, 95% CI [− 40.64 to − 18.02], *P* < 0.001) (Fig. 2c).

##### CSF protein concentration

A random-effects model was also used to analyze this variable due to high heterogeneity. The pooled CSF protein concentrations were 1.07 mg/L (95% CI 0.88 – 1.72) after IDI and 1.99 mg/L (95% CI 1.6 – 2.38) after C anti-TB. Meta-analysis revealed that the CSF protein concentration was significantly lower in the IDI group compared with the C anti-TB group (WMD − 0.79, 95%CI [− 0.96 to − 0.61], *P* < 0.001) (Fig. 2d).

#### Secondary outcome indicators: CSF findings and normalization of clinical signs and symptoms

##### CSF glucose

**Fig. 3:**
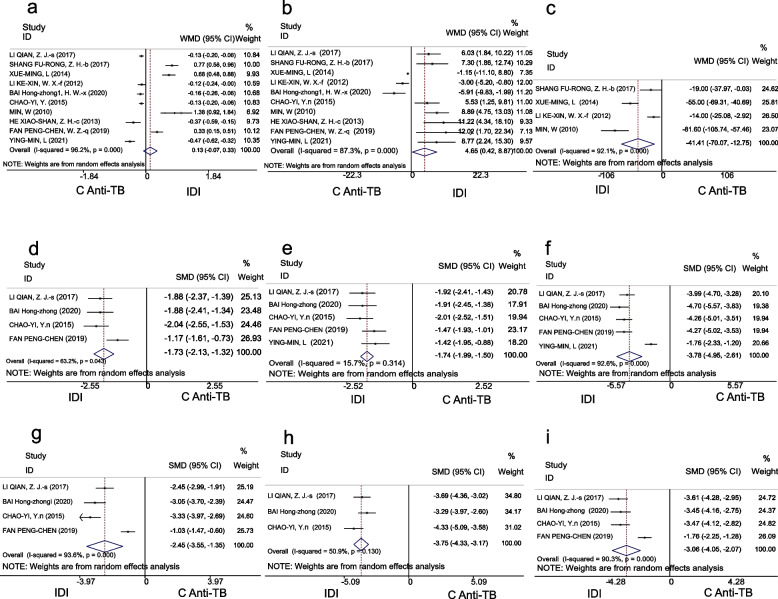
Forest plot of secondary outcomes in the IDI and C anti-TB groups, **a**-**c** Forest plot of WMD of CSF glucose, CSF chlorides, and CSF pressure. **d**-**f** Forest plot of SMD of the recovery time of CSF leukocytes, CSF protein concentration, and CSF pressure. **g**-**i** Forest plot of SMD of the recovery time of fever, coma, and headache

Due to high heterogeneity, a random-effects model was selected for the analysis of this indicator. CSF glucose concentrations in the IDI and C anti-TB groups were 2.05 mmol/L (95% CI 1.69–2.42) and 1.89 mmol/L (95% CI 1.62–2.15), respectively. However, the meta-analysis revealed that there was no significant difference between the IDI and C anti-TB groups in terms of CSF glucose enhancement (WMD 0.13, 95% CI [− 0.07 to 0.33], *P* = 0.20) (Fig. 3a).

##### CSF chlorides

Levels of CSF chlorides were 115.29 mmol/L (95%CI 110.2–120.39) in the IDI group and 110.68 mmol/L (95%CI 106.04–115.32) in the C anti-TB group. Meta-analysis revealed that the IDI group showed significant improvement in CSF chlorides indicators compared with the control group (WMD 4.65, 95% CI 0.42–8.87, *P* = 0.031) (Fig. 3b).

##### CSF pressure

A random-effects model was selected for analysis of this indicator due to high heterogeneity. Significant differences in CSF pressure were found between the IDI group (170.25 mmH_2_O) and the C anti-TB group (210.52 mmH_2_O) (WMD − 41.41, 95% CI [− 70.7 to − 12.75], *P* = 0.005) (Fig. 3c).

#### Normalization of CSF findings and clinical signs and symptoms

Recovery time indicators (CSF leukocytes, CSF protein, CSF pressure, fever, coma, and headache) were not analyzed in a single-arm meta-analysis due to inconsistent time units (days vs. hours). However, a meta-analysis showed that the recovery time for all the aforementioned indicators in the IDI groups was shorter than that for the C anti-TB group. The results for each indicator were highly significant, as follows: CSF leukocytes (SMD − 1.73, 95% CI [− 2.31 to − 1.32], *P* < 0.001), CSF protein (SMD -1.74, 95% CI [− 1.99 to − 1.5], *P* < 0.001), CSF pressure (SMD − 3.78, 95% CI [− 4.95 to − 2.61], *P* < 0.001), fever (SMD − 2.45, 95% CI [− 3.55 to − 1.35], *P* < 0.001), coma (SMD − 3.75, 95% CI [− 4.33 to − 3.17], *P* < 0.001), and headache (SMD − 3.06, 95% CI [− 4.05 to − 2.07], *P* < 0.001) (Fig. 3d-i).

### Results of higher-dose IDI group vs. lower-dose IDI group

#### Primary outcome indicators: efficacy, safety, and CSF findings

##### Treatment efficacy

**Fig. 4:**
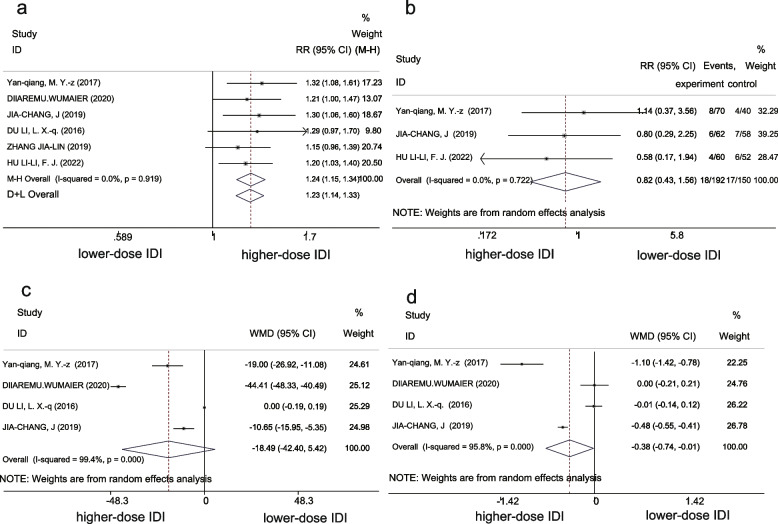
Forest plot of primary outcomes in higher-dose IDI and lower-dose IDI groups. **a** RR of the effective rate. **b** RR of adverse reaction rate. **c** WMD of CSF leukocytes. **d** WMD of CSF protein concentration

Efficacy was 93% (95%CI 90–96) and 73% (95% CI 68–79) in the higher-dose IDI and lower-dose IDI groups, respectively. Meta-analysis showed that treatment efficacy for patients receiving higher IDI doses was significantly higher than those receiving lower IDI doses (RR 1.23, 95% CI 1.14-1.33, *P* < 0.001). No heterogeneity was detected in the results (I^2^ = 0, *P* = 0.919) (Fig. 4a).

##### Adverse reactions

The adverse reaction rates were 9% (95% CI 5–13) in the higher-dose IDI group and 11% (95% CI 6–16) in the lower-dose IDI group. There was no significant difference in reported adverse reactions between the two groups according to meta-analysis (RR 0.82, 95%CI 0.43–1.56, *P* = 0.544); heterogeneity, (I^2^ = 0, *P* = 0.722) (Fig. 4b).

##### CSF leukocytes 

CSF leukocytes were 90.17 × 10^6^/L (95% CI 16.01–164.33) and 108.69 × 10^6^/L (95% CI 13.10-204.27) in the higher-dose IDI and lower-dose IDI groups, respectively. However, there was no significant difference in these values between the two groups (WMD − 18.49, 95% CI [− 42.40 5.42], *P* = 0.13) (Fig. 4c).

##### CSF protein concentration

CSF protein concentrations were 1.44 mg/L (95% CI 0.55-2.33) and 1.83 mg/L (95% CI 0.94-2.73) in the higher-dose IDI and lower-dose IDI groups, respectively. There was a minimal difference in this parameter between the two groups (WMD − 0.38, 95%CI [− 0.74 to − 0.01], *P* = 0.041) (Fig. 4d).

#### Secondary outcome indicators: CSF findings and normalization of clinical signs and symptoms

##### CSF glucose

**Fig. 5:**
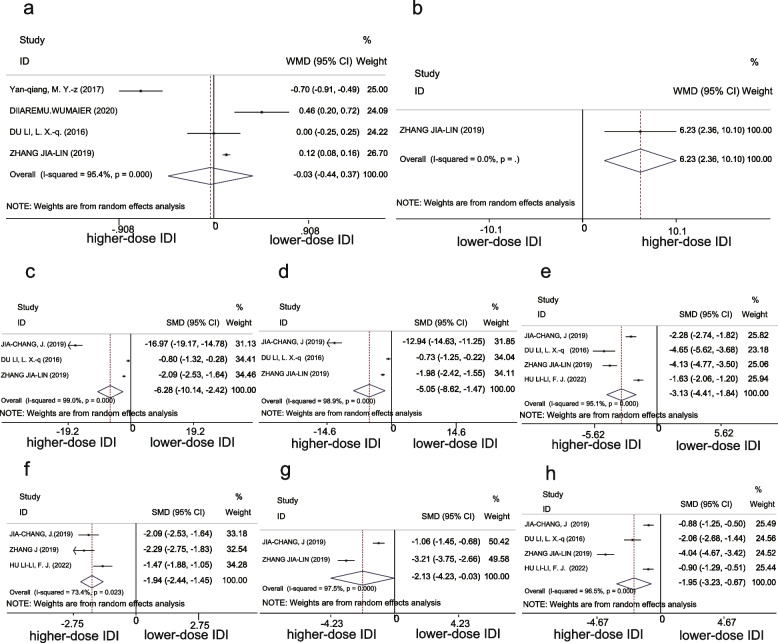
Forest plot of secondary outcomes in higher-dose IDI and lower-dose IDI groups, **a**, **b** Forest plot of WMD of CSF glucose, and CSF chlorides. **c**–**e** Forest plot of SMD of the recovery time of CSF leukocytes, CSF protein concentration, and CSF pressure. **f**-**h** Forest plot of SMD of the recovery time of fever, coma, and headache

CSF glucose concentrations were 1.97 mmol/L (95% CI 1.41–2.52) and 2.0 mmol/L (95% CI 1.47-2.54) in the higher-dose IDI and lower-dose IDI groups, respectively. There was no significant difference between the two groups in this regard (WMD − 0.03, 95% CI [− 0.44 to 0.37], *P* = 0.877) (Fig. 5a).

##### CSF chlorides

CSF chlorides were analyzed in only one study [42], and meta-analysis indicated that the improvement in CSF chlorides for the higher-dose IDI group was more significant than the lower-dose IDI group (WMD 6.23, 95% CI [2.36–10.1], *P* = 0.002) (Fig. 5b).

##### CSF pressure

Only one of the six studies included in the meta-analysis recorded CSF pressure [41]. In this study, the mean ± SD for CSF pressure following 20-days treatment with higher-dose (isoniazid 200 mg + dexamethasone 10 mg) IDI injection was 160 ± 38, and the lower-dose (isoniazid 100 mg + dexamethasone 5 mg) group (230 ± 67) (*P* < 0.05).

#### Normalization of CSF findings and clinical signs and symptoms

Recovery times for all indicators (CSF leukocytes, CSF protein, CSF pressure, fever, coma, and headache) in the higher-dose were shorter than those in the lower-dose IDI group. The results for each indicator was significant, as follows: CSF leukocytes (SMD − 6.28, 95% CI [− 10.14 to − 2.42], *P* < 0.001), CSF protein (SMD − 5.05, 95% CI [− 8.62 to − 1.47], *P* = 0.006), CSF pressure (SMD − 3.13, 95% CI [− 4.41 to − 1.84], *P* < 0.001), fever (SMD − 1.94, 95% CI [− 2.44 to − 1.45], *P* < 0.001), coma (SMD − 2.13,95% CI [− 4.23 to − 0.03], *P* = 0.047), and headache (SMD − 1.95,95% CI [− 3.23 to − 0.67], *P* = 0.003) (Fig. 5c-h).

#### Subgroup analysis

Subgroup analyses were performed on the 11 studies according to the dose of intrathecal isoniazid (100 mg vs. 50 mg).

#### Primary outcome indicators: efficacy, safety, and CSF findings

**Fig. 6:**
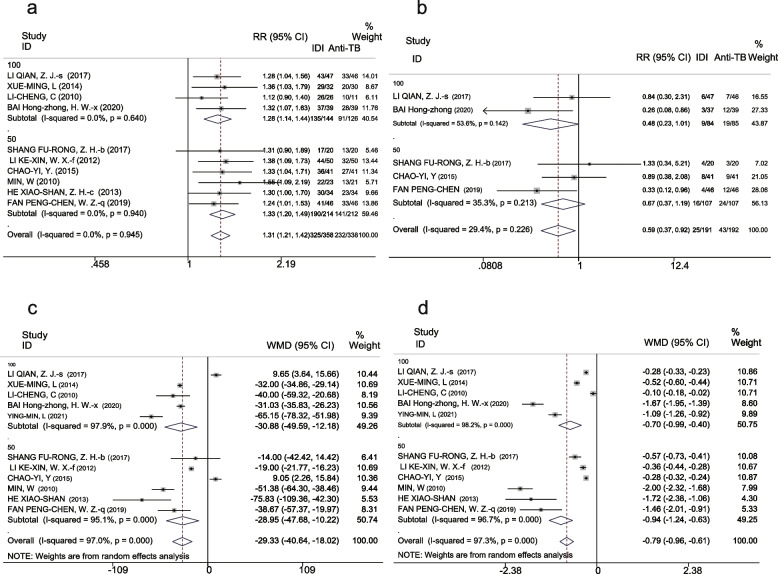
Forest plot of subgroup analysis on the primary outcomes, (**a**) Effective rate. **b** Adverse reaction rate. **c** CSF leukocytes. **d** CSF protein concentration

**Fig. 7:**
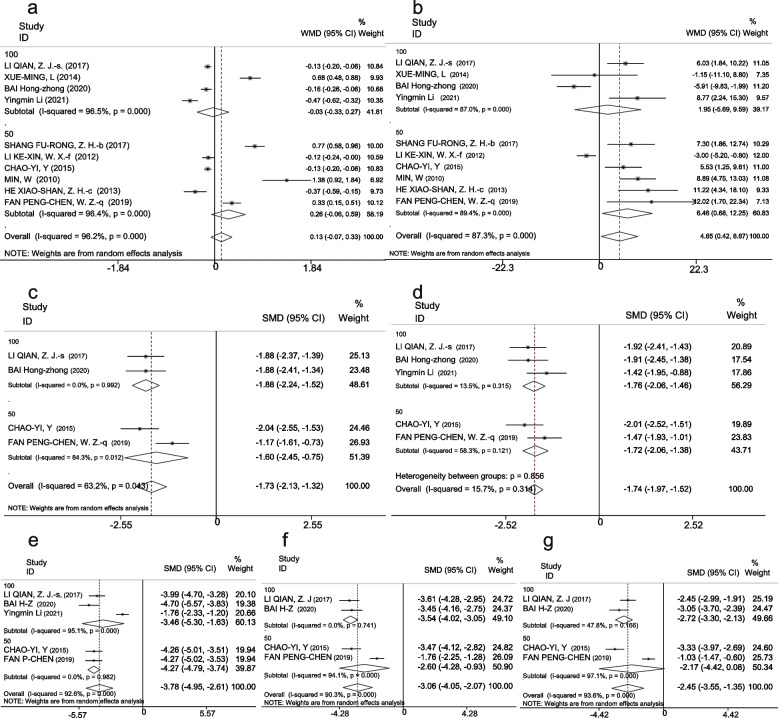
Forest plot of subgroup analysis on the secondary outcomes. **a** CSF glucose, (**b**) CSF chlorides. **c**-**g** Subgroup analysis of the recovery time of CSF leukocytes, CSF protein, CSF pressure, headache, and fever

For the 100-mg group, treatment efficacy was statistically significant (RR 1.28, 95% CI 1.14 – 1.44, *P* < 0.001), reports of adverse reactions were not different (RR 0.48, 95% CI 0.23 – 1.01, *P* = 0.053) (Fig. 6a, b). Statistically significant indicators in the 100-mg group included CSF leukocytes (WMD − 30.88, 95% CI [− 49.59 to − 12.18], *P* < 0.001) and CSF protein (WMD − 0.7, 95% CI [− 0.99 to − 0.40], *P* < 0.001) (Fig. 6c, d). The CSF glucose (WMD − 0.03, 95% CI [− 0.33 0.27], *P* = 0.848) and CSF chlorides (WMD 1.95, 95% CI [− 5.69 9.59], *P* = 0.617) were not statistically significant (Fig. 7a, b).

#### Secondary outcome indicators: CSF findings and normalization of clinical signs and symptoms

To estimate recovery time indicators (i.e., CSF leukocytes, CSF protein, CSF pressure, headache, and fever) (coma was not analyzed as a subgroup due to limited data), we conducted a related analysis. There were highly significant differences in both high- and low-IDI dose groups. In terms of the time to return to normal activities, the results for each indicator in the 100-mg group were as follows: CSF leukocytes (SMD −1.88, 95% CI [−2.24 to −1.52], *P* < 0.001), CSF proteins (SMD−1.76, 95% CI [−2.06 to −1.46], *P* < 0.001), CSF pressure (SMD −3.46, 95% CI [−5.30 to −1.63], *P* < 0.001), headache (SMD −3.54, 95% CI [− 4.02 to −3.05], *P* < 0.001), and fever (SMD −2.72, 95% CI [−3.30 to −2.13], *P* < 0.001).

For the 50-mg group, significant differences were found as follows: CSF leukocytes (SMD −1.60, 95% CI [−2.45 to −0.75], *P* < 0.001), CSF protein (SMD − 1.72, 95% CI [−2.06 to − 1.38], *P* < 0.001), CSF pressure (SMD −4.27, 95% CI [−4.79 to −3.74], *P* < 0.001), and headache (SMD −2.60, 95% CI [−4.28 to −0.93], *P* = 0.002). Fever (SMD −2.17, 95% CI [−4.42 to 0.08], *P* = 0.059) was not statistically significant (Fig. 7c-g).

#### Publication bias and sensitivity analysis

The funnel plot showed a symmetrical distribution of included studies. Harbord, Peters, and Egger’s tests indicated that there was no potential publication bias among the primary indicators (Additional files 3 and 4). Sensitivity analyses confirmed the robustness of the results (Additional files 5 and 6).

## Discussion

The present study primarily investigated the therapeutic efficacy and safety of IDI at varying concentrations, in conjunction with standard oral or intravenous anti-TB treatment, in adult patients with TBM. Our findings indicated that the combination therapy significantly improved clinical outcomes and decreased adverse reaction rate compared to C anti-TB alone, which was consistent with previous studies [12, 13]. Furthermore, the safety profile of the combined therapy was acceptable, with no significant increase in adverse events, suggesting that this approach could be a viable option for improving TBM treatment outcomes.

The primary innovation of our study was the comprehensive evaluation of IDI + C anti-TB therapy vs. C anti-TB therapy only in adult patients with TBM. This study filled an important knowledge gap and provided strong evidence of the efficacy and safety of IDI, which has not been extensively studied in the previous literature. Our study uniquely integrated these treatments and evaluated their synergistic effects. The results demonstrate that adjunct IDI treatment groups showed greater treatment efficacy, highlighting the potential of IDI to enhance therapeutic outcomes, compared to patients receiving C anti-TB treatment strategies. Furthermore, the lower incidence of adverse reactions in the IDI group underscored the safety of this combined approach. These findings were particularly novel as they provided a new therapeutic strategy that could potentially improve the prognosis of TBM patients, a population that had historically faced high morbidity and mortality rates.

Moreover, the significant reduction in CSF leukocytes and protein concentrations in the IDI group indicated a more effective inflammatory response control, which were crucial in TBM treatment [7]. The observed improvements in CSF chloride levels and a shorter recovery time of CSF findings and clinical signs and symptoms (Fig. 3d-i) further supported the therapeutic benefits of IDI, suggesting enhanced CSF homeostasis.

Findings of the meta-analysis of higher-dose vs. lower-dose adjuvant IDI and the subgroup analysis of IDI + C anti-TB therapy vs. C anti-TB therapy only showed a similar trend in terms of primary indicators: the efficacy of the higher-dose IDI group was significantly higher than that of the lower-dose IDI group. While there was no difference between the groups about reported adverse reactions. By conducting a thorough meta-analysis and subgroup analysis, this study offered a detailed understanding of the dose-dependent effects of IDI, further contributing to the optimization of treatment protocols for TBM.

### Adjunct intrathecal IDI for TBM

The intrathecal injection is the process of injecting a drug directly into the subarachnoid space [10, 44]. As such, the drug enters the CSF directly without crossing the blood–brain barrier and reaches its effective concentration rapidly [10, 44]. Advantages of intrathecal therapy include increased concentrations of anti-TB drugs in the CSF to improve efficacy, and reduced side effects of anti-TB drugs, such as gastrointestinal discomfort and hepatotoxicity [10, 44, 45]. However, intrathecal therapy still carries risks, such as nosocomial CNS infections even when injections are performed under sterile conditions [46, 47]. Currently, there is no consensus on intrathecal injection frequency and safety, and there are fewer references on intrathecal injections for TBM [10] (the frequency of intrathecal injections for each of the included studies is found in Table 2).

### Evidence of treatment efficacy and adverse reactions

Due to the lack of high-quality RCTs on intrathecal injection for TBM treatment, very little is known about its efficacy [10]. As early as 1981 P Dajez et al. reported a successful case of TBM treated by intraventricular administration of rifampicin [48]. One study also reported a case of a patient with severe TBM successfully treated with intraventricular rifampicin administration for 50 consecutive days without local or systemic side effects [49]. A study from China reported the successful treatment by intrathecal injection of isoniazid, dexamethasone, and chymotrypsin in two cases of multiple brain tuberculomas that developed after in vitro fertilization, embryo transfer, and without reporting adverse reactions, when C anti-TB treatment was ineffective [12]. A recent study on refractory TBM has shown that intrathecal and intracerebroventricular drug delivery has been identified as a key method that can overcome the difficulties posed by the blood-brain barrier and increase drug levels within the CSF, which not only helps to increase anti-TB drug concentrations in the CSF but also mitigates both drug resistance and the risk of disease relapse [10]. However, the intrathecal injection method is not currently included in key guidelines for treatment management of TBM, and no standardized dosage and administration method is available [10]. The intrathecal injection can be regarded as an adjunctive host-directed therapy [10].

### Basis for selection of CSF primary indicators

CSF leukocytes, total protein, and glucose levels are important parameters for TBM [50]. Some studies have concluded that increased CSF total protein, decreased CSF-to-serum glucose ratio, and increased CSF leukocytes with lymphocytes are characteristics of TBM [50, 51]. Others have concluded that increased CSF leukocytes and protein, and decreased CSF glucose are features of TBM [17]. It is generally accepted that the key CSF features of TBM: CSF Leukocytes < 1000 cells per mm^3^, CSF Protein > 100 mg/dl, and CSF: blood glucose ratio < 0.5 [7]. Unfortunately, the CSF-to-serum glucose ratio, an index that was not available in our included studies. As shown here, the above studies consistently concluded that elevated CSF leukocytes and total protein are important features of TBM [17, 50, 51]. Therefore, we selected CSF leukocytes, and CSF protein as our primary outcomes.

### Limitations

There was significant heterogeneity in the quantitative data used in this study, possibly attributed to the different age distributions across the studies and the limited number of studies in the present literature. Furthermore, we have only analyzed the clinical symptoms of fever, headache, and coma. Due to the limited amount of available data, the accuracy of these results needs to be further verified. In addition, symptoms such as nausea, vomiting, and neck stiffness associated with meningeal infection have not been studied. Heterogeneity in the frequency of intrathecal injection across individual studies may also impact the results. Finally, the studies included were mostly from China, which will likely impact the generalizability of the results. It is also essential to consider the variability in IDI dosages, which may affect the generalizability of the results. Future research should focus on standardizing IDI dosages and conducting large-scale, multi-center trials to validate these findings and further elucidate the long-term benefits and safety of IDI in TBM treatment.

## Conclusions

The findings suggest that adjunct IDI can be effectively integrated into the treatment regimen, potentially reducing inflammation and improving patient prognosis compared to conventional oral or intravenous anti-TB medication. Further, large-scale RCTs are needed. The comprehensive analysis of the existing RCT literature included in this meta-analysis may provide a reference point for clinical TBM treatment and future studies. Further investigations into the optimal dosing strategies for IDI are also warranted to maximize therapeutic benefits while minimizing potential adverse effects.

### Supplementary Information

Supplementary Material 1. 

Supplementary Material 2.

Supplementary Material 3.

Supplementary Material 4.

Supplementary Material 5.

Supplementary Material 6.

## Data Availability

The datasets used and analyzed during the current study are available from the corresponding author upon reasonable request.

## Abbreviations

IDI: Intrathecal dexamethasone and isoniazid
TBM: Tuberculous meningitis
TB: Tuberculosis
C anti-TB: Conventional anti-tuberculosis
CNS: Central nervous system
CSF: Cerebrospinal fluid
CNKI: Chinese National Knowledge Infrastructure
CBM: Chinese Biomedical Literature Database
VIP: Chinese Scientific Journal Database
WMD: Weighted mean difference
SMD: Standardized mean difference
RCT: Randomized controlled trial
RR: Risk ratio
95% CIs: 95% Confidence intervals
