# Supplementary material for: Efficacy and safety of intrathecal dexamethasone combined with isoniazid in the treatment of tuberculous meningitis: a meta-analysis
Source: BMC Neurol. 2024 Jun 10;24:194. doi: 10.1186/s12883-024-03701-4 (PMC11163761; doi:10.1186/s12883-024-03701-4)
Supplement: Supplementary file 2 — Supplementary Material 2. [file 12883_2024_3701_MOESM2_ESM.pdf]

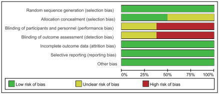

Assessment of publication bias of included studies

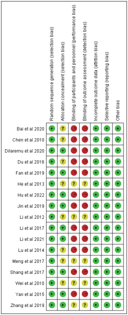

Quality assessment of Included Studies
