# Supplementary material for: Efficacy and safety of intrathecal dexamethasone combined with isoniazid in the treatment of tuberculous meningitis: a meta-analysis
Source: BMC Neurol. 2024 Jun 10;24:194. doi: 10.1186/s12883-024-03701-4 (PMC11163761; doi:10.1186/s12883-024-03701-4)
Supplement: Supplementary file 3 — Supplementary Material 3. [file 12883_2024_3701_MOESM3_ESM.pdf]

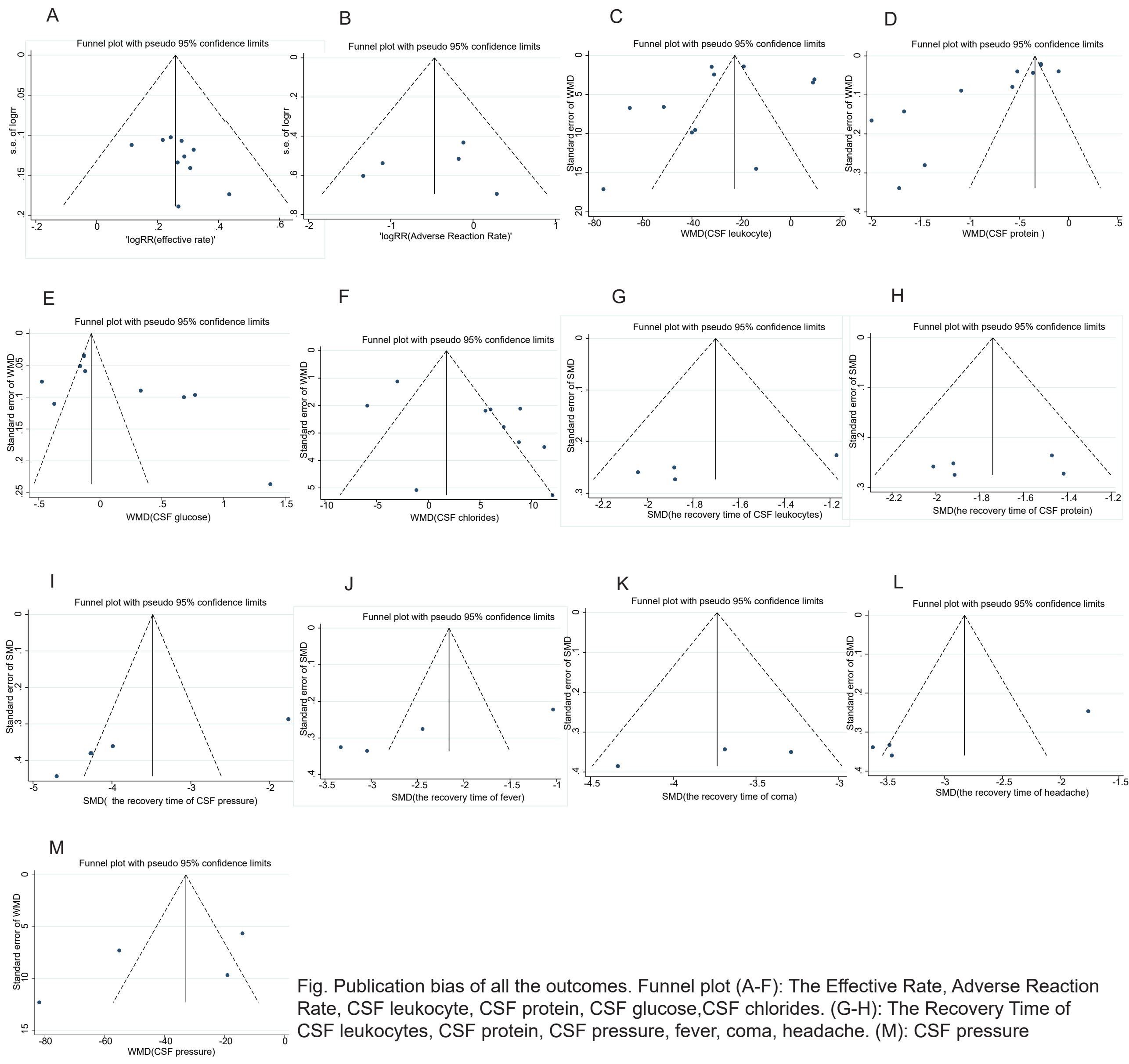

Fig. Publication bias of all the outcomes. Funnel plot (A-F): The Effective Rate, Adverse Reaction Rate, CSF leukocyte, CSF protein, CSF glucose,CSF chlorides. (G-H): The Recovery Time of CSF leukocytes, CSF protein, CSF pressure, fever, coma, headache. (M): CSF pressure
