# Supplementary material for: Efficacy and safety of intrathecal dexamethasone combined with isoniazid in the treatment of tuberculous meningitis: a meta-analysis
Source: BMC Neurol. 2024 Jun 10;24:194. doi: 10.1186/s12883-024-03701-4 (PMC11163761; doi:10.1186/s12883-024-03701-4)
Supplement: Supplementary file 4 — Supplementary Material 4. [file 12883_2024_3701_MOESM4_ESM.pdf]

Table A quantitative assessment for publication bias

Quantitative Assessment for Publication Bias	The Effective Rate	Adverse Reaction Rate	CSF leukocyte	CSF protein	CSF glucose	CSF chloride	CSF pressure
Number of studies	10	5	11	11	10	10	4
P value for Harbord tests	0.028	0.369	-	-	-	-	-
P value for peters tests	0.391	0.477	-	-	-	-	-
P value for Egger tests	-	-	0.743	0.002	0.081	0.048	0.350

Table B quantitative assessment for publication bias

Quantitative Assessment for Publication Bias						
The recovery time of indicators	CSF leukocytes	CSF protein	CSF pressure	fever	coma	headache
Number of studies	4	5	5	4	3	4
P value for Egger tests	0.126	0.693	0.012	0.025	0.367	0.024

Table (Online Supplementary) Single-arm meta-analysis of outcomes

	Outcomes	
	IDI	CAnti-TB
The effective rate	RR 91% (95%CI 88–94)	RR 70% (95% CI 65–75)
Adverse reaction rate	RR 12% (95%CI 7–16)	RR 21% (95% CI 15–27)
CSF leukocytes	WMD 110.05×10^6/L (95%CI 65.55–154.56)	WMD 141.79×10^6/L (95% CI 94.31–189.27)
CSF protein	WMD 1.07mg/L (95%CI 0.88–1.72)	WMD 1.99mg/L (95% CI 1.6–2.38)
CSF glucose	WMD 2.05mmol/L (95%CI 1.69–2.42)	WMD 1.89mmol/L (95%CI 1.62–2.15)
CSF chlorides	WMD 115.29mmol/L (95%CI 110.2–120.39)	WMD 110.68mmol/L (95%CI 106.04–115.32)

IDI, intrathecal dexamethasone and isoniazid; CAnti-TB, conventional anti-tuberculosis
