# Supplementary material for: Efficacy and safety of intrathecal dexamethasone combined with isoniazid in the treatment of tuberculous meningitis: a meta-analysis
Source: BMC Neurol. 2024 Jun 10;24:194. doi: 10.1186/s12883-024-03701-4 (PMC11163761; doi:10.1186/s12883-024-03701-4)
Supplement: Supplementary file 5 — Supplementary Material 5. [file 12883_2024_3701_MOESM5_ESM.pdf]

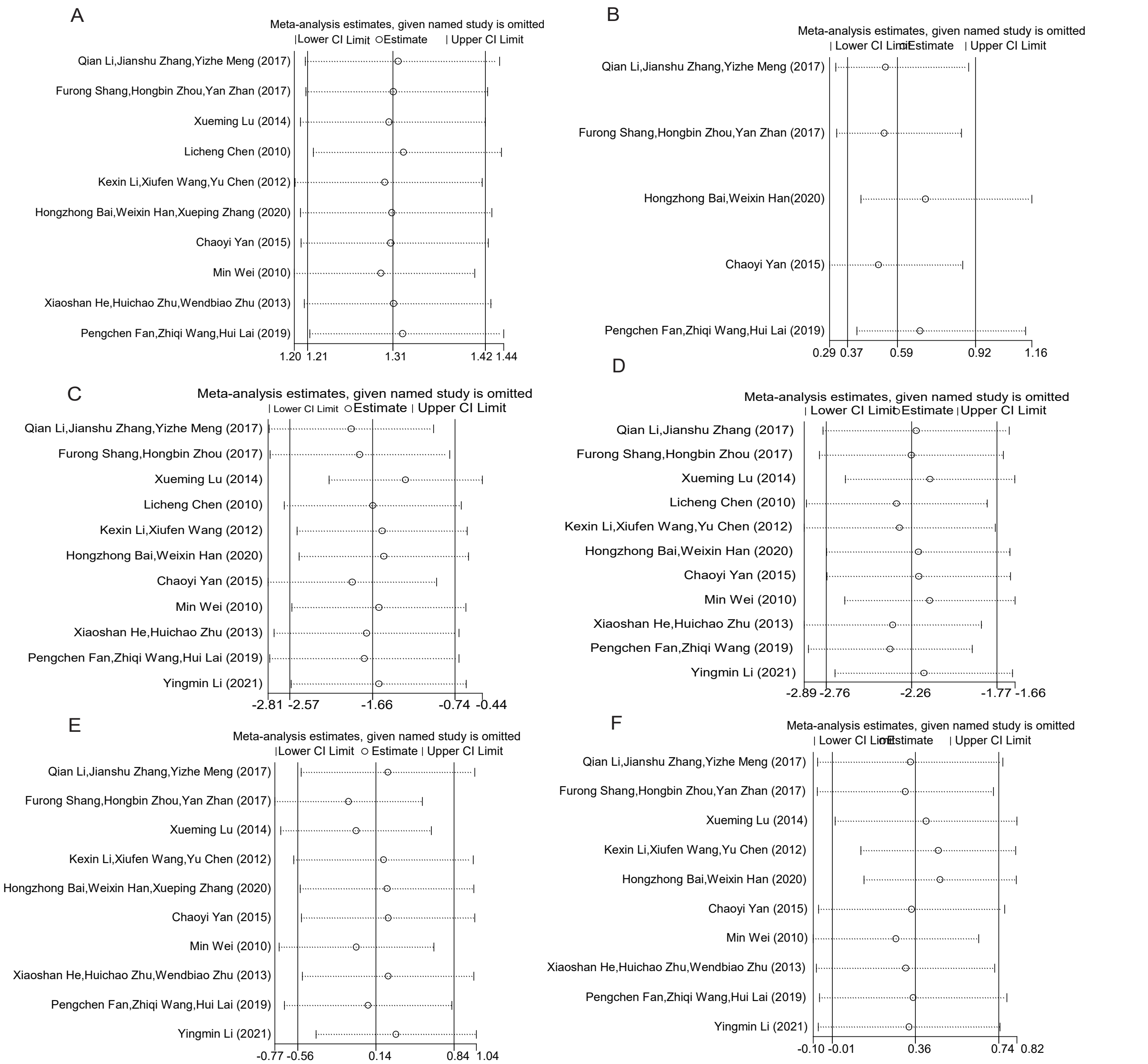

Fig. Sensitivity of all the outcomes. (A) the effective rate. (B) adverse reaction rate. (C) CSF leukocytes. (D) CSF protein. (E) CSF glucose.(F)CSF chlorides.
