# Supplementary material for: Efficacy and safety of intrathecal dexamethasone combined with isoniazid in the treatment of tuberculous meningitis: a meta-analysis
Source: BMC Neurol. 2024 Jun 10;24:194. doi: 10.1186/s12883-024-03701-4 (PMC11163761; doi:10.1186/s12883-024-03701-4)
Supplement: Supplementary file 6 — Supplementary Material 6. [file 12883_2024_3701_MOESM6_ESM.pdf]

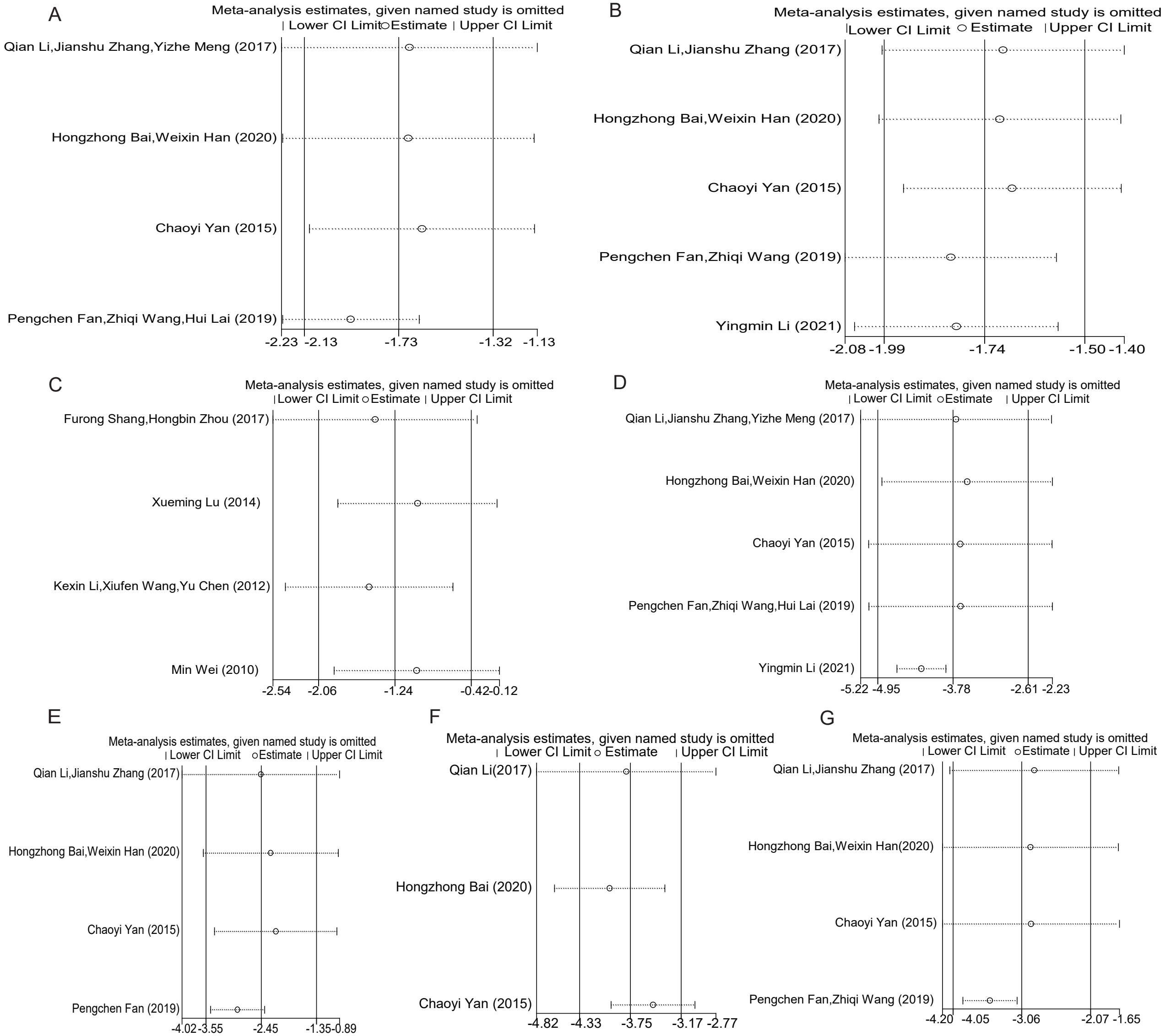

Fig. Sensitivity of all the outcomes. (A) the recovery time of CSF leukocytes. (B) the recovery time of CSF proteins. (C) CSF pressure. (D) the recovery time of CSF pressure . (E) the recovery time of fever.(F)the recovery time of coma. (G) the recovery time of headache
